# Effects of *TPMT, NUDT15*, and *ITPA* Genetic Variants on 6-Mercaptopurine Toxicity for Pediatric Patients With Acute Lymphoblastic Leukemia in Yunnan of China

**DOI:** 10.3389/fped.2021.719803

**Published:** 2021-10-01

**Authors:** Xiaoyan Mao, Runxiu Yin, Gaoyuan Sun, Yan Zhou, Chunhui Yang, Chunlian Fang, Yuhong Wu, Tingting Cui, Li Liu, Jiaxin Gan, Xin Tian

**Affiliations:** ^1^Department of Hematology, The Affiliated Children's Hospital of Kunming Medical University, Kunming Medical University, Kunming, China; ^2^Department of Pediatrics, Sichuan Clinical Research Center for Birth Defects, The Affiliated Hospital of Southwest Medical University, Luzhou, China; ^3^Department of Pediatric Hematology, Dali University, Dali, China

**Keywords:** *TPMT*, *NUDT15*, *ITPA*, 6-mercaptopurine, pediatric patients, acute lymphoblastic leukemia

## Abstract

**Background:** 6-Mercaptopurine (6-MP) is the cornerstone of current antileukemia regimen and contributes greatly to improve the survival of pediatric acute lymphoblastic leukemia (ALL) patients. However, 6-MP dose-related toxicities limit its application. *TPMT, NUDT15*, and *ITPA* are pharmacogenetic markers predicting 6-MP-related toxicities, but their genetic polymorphisms differ from those of ethnic populations. In Yunnan province, a multiethnic region of China, we had no genetic data to predict 6-MP toxicities. In this study, we evaluated the most common variants involved in 6-MP metabolism—TPMT^*^3C (rs1142345), *NUDT15* c.415C>T (rs116855232), and *ITPA* c.94C>A (rs1127354) variants—in our cohort of pediatric ALL patients.

**Methods:** A total of 149 pediatric ALL patients in the Affiliated Children's Hospital of Kunming Medical University (Yunnan Children's Medical Center) from 2017 to 2019 were enrolled in this retrospective study. We assessed the TPMT^*^3C (rs1142345), *NUDT15* c.415C>T (rs116855232), and *ITPA* c.94C>A (rs1127354) frequencies and evaluated association between genotypes and 6-MP toxicities, 6-MP dose, and event-free survival (EFS) in these ALL patients.

**Results:** The allele frequencies of TPMT^*^3C (rs1142345), *NUDT15* c.415C>T (rs116855232), and *ITPA* c.94C>A (rs1127354) were 1.34%, 14.43%, and 18.79%, respectively. Only *NUDT15* c.415C>T (rs116855232) was strongly associated with 6-MP toxicity and 6-MP tolerable dose. *NUDT15* c.415C>T was related to leukopenia, *p* = 0.008, OR = 2.743 (95% CI: 1.305–5.768). The T allele was significantly correlated with 6-MP tolerable dose, dose of *NUDT15* c.415C>T wild genotype CC 39.80 ± 1.32 mg/m^2^, heterozygotes CT 35.20 ± 2.29 mg/m^2^, and homozygotes TT 18.95 ± 3.95 mg/m^2^. 6-MP tolerable dose between CC and TT had a significant difference, *p* = 0.009. Between CC and CT, and CT and TT, they had no significant difference. EFS showed no significant difference among *NUDT15* c.415C>T genotypes.

**Conclusion:** *NUDT15* c.415C>T (rs116855232) was an optimal predictor for 6-MP toxicity and tolerable dose in pediatric ALL patients from Yunnan province, a multiethnic region in China, and would play an important role in precise therapy for ALL.

## Introduction

Acute lymphoblastic leukemia (ALL) is the most common pediatric malignant cancer. Chemotherapy remains the major treatment, including induction, consolidation, and maintenance, with overall survival rate of 80–90% (1–3). 6-Mercaptopurine (6-MP), one of purine antimetabolites, is the cornerstone of current antileukemia regimen. Especially in maintenance therapy, 6-MP and methotrexate (MTX) are primary components and play important roles in long-term remission (4). In a clinic setting, 6-MP has severe toxicities including myelosuppression, hepatotoxicity, gastrointestinal distress, and alopecia. In particular, myelosuppression is complicated with severe infection, leading to drug reduction or withdrawal, even treatment interruption, and patient death (5).

Thiopurine methyltransferase (TPMT) is the first described enzyme linked to 6-MP intolerance, and its decreased enzyme activity is related to increased drug toxicities (6). Three *TPMT* genetic polymorphisms (^*^2, ^*^3A, and ^*^3C) account for 95% low enzyme activity (7), but *TPMT* genetic polymorphisms cannot explain all 6-MP intolerance. In fact, the frequency of *TPMT* genetic polymorphism differs among ethnic groups: high in European population but low in Asian population (8). In China, total frequency of variant *TPMT* alleles is 2.91%, and the most common genetic polymorphism is ^*^3C, differing between ethnic groups (9).

Nucleoside diphosphate-linked moiety X-type motif 15 (NUDT15) is a newly found important enzyme associated with 6-MP metabolism. In 2014, Yang et al. first identified the relationship between *NUDT15* genetic polymorphism and myelosuppression caused by thioprine drugs (10). They reported *NUDT15* c.415 C>T (rs116855232) linked to thiopurine-induced leukopenia in patients with inflammatory bowel disease (IBD), and the variant frequency of *NUDT15* is much higher than that of *TPMT* in Korean population. The updated guideline from Clinical Pharmacogenetics Implementation Consortium Guideline for Thiopurine Dosing Based on *TPMT* and *NUDT15* Genotypes included nine *NUDT15* single-nucleotide polymorphisms (SNPs) (8), and the most frequent variant is *NUDT15* ^*^3, rs116855232 (c.415C>T; p.R139C). *NUDT15* genetic polymorphisms are variable across different ethnic populations. In contrast to *TPMT* gene, *NUDT15* variation is rarely found in European and African populations as compared with Asian population (11). In China, the frequency of variant *NUDT15* is higher than that of *TPMT*, and *NUDT15* polymorphisms are responsible for 6-MP intolerance (12, 13).

Inosine triphosphate (ITPA) is also related to thioprine metabolism. In 2004, Mariaki et al. reported the variant of *ITPA* c.94C>A inducing decreased enzyme activity, thus causing azathioprine intolerance. It first confirmed the association between *ITPA* genetic variant and thioprine drug intolerance (14). *ITPA* c.94C>A is one of the most frequent variants decreasing enzyme activity, and variant frequency also differs between ethnic groups. Data from the National Institutes of Health (NIH) show the frequency of *ITPA* c.94C>A in Asia population of 18.5%, European population of 7.2%, and African population of 5.3%. In a clinic setting, the relationship between *ITPA* genetic polymorphisms and thioprine toxicity has not been unified, especially in different ethnic populations (15–17).

*TPMT, NUDT15*, and *ITPA* genetic polymorphisms associate with thioprine toxicity; and their frequencies of variants differ among ethnic populations. In Asia, China has 56 ethnic groups, and Yunnan province is a multiethnic region with 25 ethnic groups. The frequencies of *TPMT, NUDT15*, and *ITPA* genetic polymorphisms for pediatric ALL patients in this area have not been reported. The aim of this study is to measure the frequencies of the most common variants involved in 6-MP metabolism—TPMT^*^3C (rs1142345), *NUDT15* c.415C>T (rs116855232), and *ITPA* c.94C>A (rs1127354)—in children with ALL from Yunnan province and to indicate whether these genetic variants could predict 6-MP toxicity and tolerable dose during ALL maintenance therapy.

## Materials and Methods

### Ethical Statement

This study was conducted in accordance with the Declaration of Helsinki guidelines and was approved by ethics committee of a children's hospital affiliated to Kunming Medical University (No. 2020-03-200-k01). Written informed consent was obtained from parents of ALL patients before this study.

### Patients and Data Collection

Children with ALL hospitalized in the Affiliated Children's Hospital of Kunming Medical University (Yunnan Children's Medical Center) from 2017 to 2019 were enrolled in this retrospective study. They received CCLG-ALL-2015 therapy, had finished at least 6 months' maintenance therapy, and were followed up until 2021 with complete material.

The maintenance phase consisted of daily oral 6-MP (50 mg/m^2^/day), weekly oral MTX (20 mg/m^2^/time), monthly intravenous vincristine (VCR; 1.5 mg/m^2^/time, <2 mg/time), and monthly oral dexamethasone (DEX; 6 mg/m^2^/day, 1–5 days). Blood count was performed once or twice a week at the beginning of maintenance treatment and then at a 2-week interval to maintain white blood cell (WBC) count of 2.0–3.0 × 10^9^/L. Leukopenia was defined as WBC count <2.0 × 10^9^/L. Liver function was performed every month, and hepatotoxicity was defined as alanine aminotransferase (ALT) or aspartate aminotransferase (AST) level >5-fold of normal.

### Genetic Analyses

DNA was extracted from 200 μl of EDTA-treated peripheral blood by paramagnetic particle method according to the instruction of nucleic acid extraction and purification kit (Huaxia, Beijing, China). TPMT^*^3C (rs1142345), *NUDT15* c.415C>T (rs116855232), and *ITPA* c.94C>A (rs1127354) were genotyped by fluorescence *in situ* hybridization using thioprine SNP locus genotype kit (Huaxia, Beijing, China). All analyses were performed in the laboratory of the Affiliated Children's Hospital of Kunming Medical University (Yunnan Children's Medical Center).

### Statistical Analysis

According to type of data, patient's characteristics were analyzed by Pearson's chi-square test, Fisher's exact test, or the Mann–Whitney *U*-test. Odds ratios and 95% confidence intervals were determined using logistic regression analysis. Receiver operating characteristic (ROC) curves were obtained to plot the sensitivity and specificity for genotypes to predict the development of leucopenia. Event-free survival (EFS) was calculated by the Kaplan–Meier method, and differences were compared using the log-rank test. All genotype frequencies were computed and tested for the Hardy–Weinberg equilibrium. All the tests were two-tailed, and probability values <0.05 were considered statistically significant. The analyses were performed using the SPSS (version 24.0), and the figures were performed by GraphPad Prism software (version 7.0).

## Results

### Baseline Characteristics of Patients and Genotype Frequencies

A total of 149 pediatric ALL patients were enrolled in this study, including 115 ethnic Han and 34 ethnic minorities. Baseline characteristics of these patients, including the specific ethnic minorities and variant frequencies of *TPMT, NUDT15*, and *ITPA*, are shown in Table 1.

**Table 1:** Baseline characteristics of patients and genotype frequencies.

**Characteristics**	
Total number of patients, n	149
**Ethnic, n (%)**	
Ethnic Han	115 (77)
Ethnic minorities	34 (23)
Ethnic Bai	1
Ethnic Dai	3
Ethnic Hani	6
Ethnic Hui	3
Ethnic Lagu	1
Ethnic Suli	1
Ethnic Miao	1
Ethnic Wa	1
Ethnic Yao	1
Ethnic Yi	14
Ethnic Zhuang	2
**Sex, n (%)**	
Female	64 (43)
Male	85 (57)
**Age at diagnosis (year), median (range)**	5.92 (0.63–13.75)
**Risk group, n (%)**	
Standard risk	33 (22.15)
Moderate risk	97 (65.10)
High risk	19 (12.75)
**Immunology type, n (%)**	
T-ALL	10 (6.71)
B-ALL	135 (90.6)
Mix type	2 (1.34)
Unclassified	2 (1.34)
**Genotype, n (%)**	
*TPMT* *3C	
TT	145 (97.32)
TC	4 (2.68)
CC	0
*NUDT15* c.415C>T (rs116855232)	
CC	109 (73.15)
CT	37 (24.83)
TT	3 (2.01)
*ITPA* c.94C>A (rs1127354)	
CC	100 (67.11)
CA	42 (28.19)
AA	7 (4.7)
TPMT*3C (TC) + *ITPA* c.94C>A (CA)	1 (0.67)
*ITPA* c.94C>A (CA) + *NUDT15* c.415C>T (CT)	10 (6.7)
Follow time (month), median (range)	18 (8–47)

*TPMT, thiopurine S-methyltransferase; NUDT15, nucleoside diphosphate-linked moiety X-type motif 15; ITPA, inosine triphosphate pyrophosphatase*.

### Frequency of *TPMT, NUDT15*, and *ITPA* Genetic Variants

For each minority, the number of patients was too little, with most minority with only one patient, so we only compare the frequency of gene variant in this region and not between ethnicities. The frequency of TPMT^*^3C in this region is 2.68% (4/149), and the allele frequency is 1.34%. The frequency of *NUDT15* c.415C>T in this region is 26.84% (40/149), and the allele frequency is 14.43%. The frequency of *ITPA* c.94C>A in this region is 32.89% (49/149), and the allele frequency is 18.79%. The frequencies of these genotypes and alleles did not deviate from the Hardy–Weinberg equilibrium (*p* > 0.05).

Allele frequency = (frequency of heterozygotes + frequency of homozygotes × 2)/(Total sample numbers × 2) × 100%.

### Associations Between Genetic Variants and 6-Mercaptopurine Toxicities

**Figure 1:**
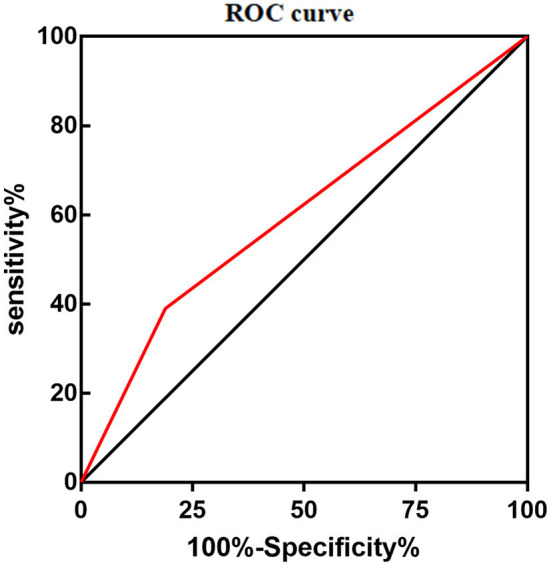
Area under the curve (AUC) of *NUDT15* c.415C > T predicting 6-MP related leucopenia was 0.600.

During maintenance therapy, leukopenia happened at 1 week after 6-MP treatment (1, 1–12 weeks), while hepatotoxicity usually at 1.5 months later (1.5 and 1–18 months). To our surprise, only the variant of *NUDT15* c.415C>T was related to leukopenia, *p* = 0.008, OR = 2.743 (95% CI: 1.305–5.768) (Table 2). The area under the curve (AUC) of *NUDT15* c.415C>T predicting leukopenia is 0.600 (Figure 1). The variants of TPMT^*^3C and *ITPA* c.94C>A were not related to leukopenia. All of the three genetic variants had no relationship with hepatotoxicity (Table 2).

**Table 2:** Associations between genetic variants and 6-MP toxicities.

**Genotype**	**Leukopenia**				**Hepatotoxicity**			
	**No**	**Yes**	***p***	**OR (95% CI)**	**No**	**Yes**	***p***	**OR (95% CI)**
TPMT*3C			0.153	–			0.583	–
TT	86	59			115	30		
TC	4	0			4	0		
*NUDT15* c.415C>T			**0.008**	**2.743** **(1.305–5.768)**			0.370	1.483 (0.625–3.621)
CC	73	36			89	20		
CT or TT	17	23			30	10		
*ITPA* c.94C>A			0.200	1.573 (0.786–3.148)			0.353	1.477 (0.646–3.379)
CC	64	36			82	18		
CA or AA	26	23			37	12	

*Leukopenia is defined as WBC count <2.0 × 10^9^/L. Hepatotoxicity is defined as ALT or AST level > 5-fold of normal*.

*6-MP, 6-mercaptopurine; WBC, white blood cell; ALT, alanine aminotransferase; AST, aspartate aminotransferase*.

### Associations Between *NUDT15* c.415C>T (rs116855232) and 6-Mercaptopurine Dose

**Figure 2:**
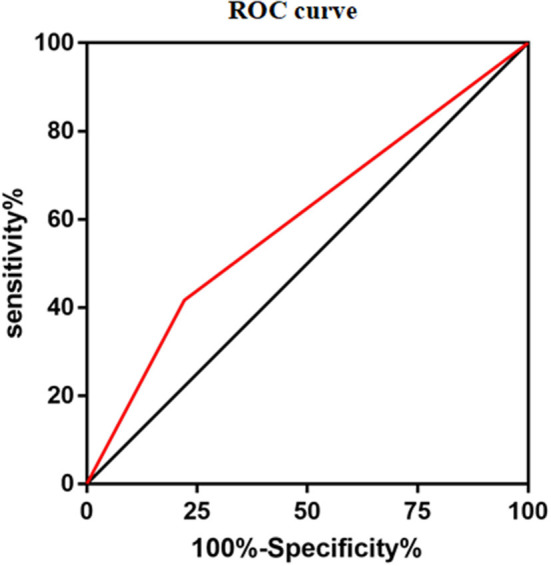
Area under the curve (AUC) of *NUDT15* c.415C > T predicting 6-MP dose decreased was 0.598.

**Figure 3:**
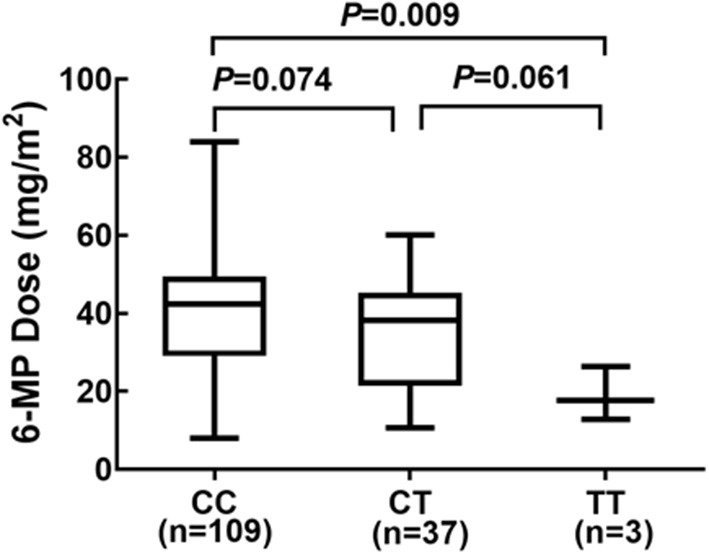
Genotype of *NUDT15* c.415C > T and 6-MP dose at maintenance period 109 patients with CC, and 6-MP dose 39.80 ± 1.32 mg/m^2^; 37 patients with CT and 6-MP dose 35.20 ± 2.29 mg/m^2^; 3 patients with TT and 6-MP dose 18.95 ± 3.95 mg/m^2^.

**Figure 4:**
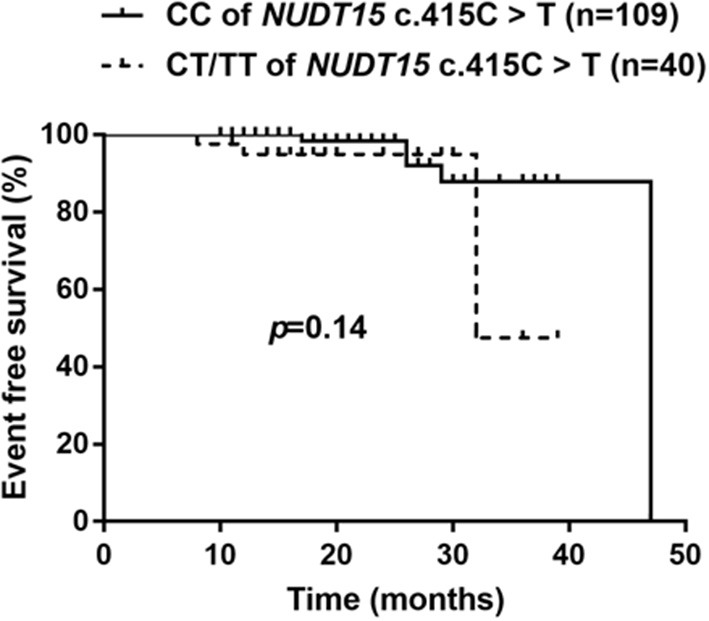
Kaplan–Meier's plots of EFS for *NUDT15* c.415C > T variants (CC and CT/TT), *P* = 0.14. In total 149 patients, followed 18 (8–47) months, *NUDT15* c.415C > T variants had no effect on long-time survival for these ALL patients in this region.

Considering *NUDT15* c.415C>T associated with leucopenia caused by 6-MP, we further found evidence that it also caused 6-MP tolerable dose decrease, *p* = 0.021, OR = 2.514 (95% CI: 1.132–5.583), and the AUC of *NUDT15* c.415C>T predicting 6-MP tolerable dose decrease was 0.598 (Figure 2). In 149 patients with ALL, 109 patients had wild *NUDT15* c.415C>T (CC), 37 patients had heterozygotes *NUDT15* c.415C>T (CT), and three patients had homozygotes *NUDT15* c.415C>T (TT). In order to evaluate effect of *NUDT15* c.415C>T on 6-MP tolerable dose, we further compared 6-MP tolerable doses for each genotype separately. To maintain WBC count of 2.0–3.0 × 10^9^/L, 6-MP tolerable dose differed among different genotypes of *NUDT15* c.415C>T, CC with 39.80 ± 1.32 mg/m^2^, CT with 35.20 ± 2.29 mg/m^2^, and TT with 18.95 ± 3.95 mg/m^2^. 6-MP tolerable dose between CC and TT had a significant difference, *p* = 0.009. While in the comparison between CC and CT, and CT and TT, they had no significant difference (Figure 3). Although variants of *NUDT15* c.415C>T may decrease 6-MP tolerable dose in maintenance therapy, EFS shows no difference between them (Figure 4).

**Figure 5:**
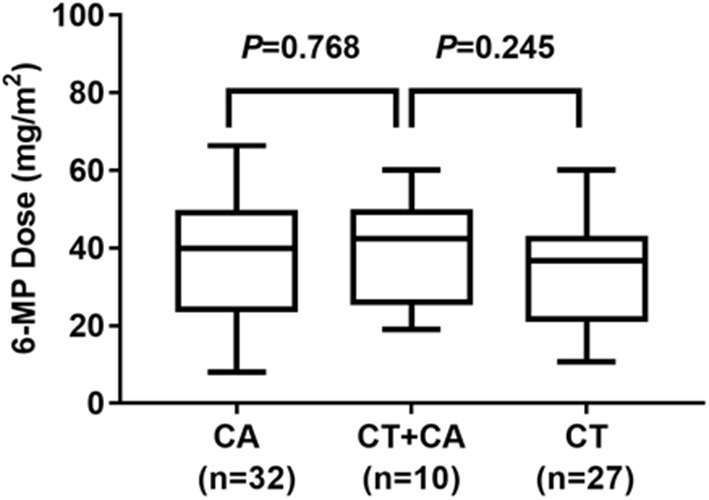
6-MP dose for patients with *ITPA* c.94C>A (CA) and *NUDT15* c.415C>T (CT) 27 patients with CT, and 6-MP dose 33.56 ± 2.70 mg/m^2^; 32 patients with CA, and 6-MP dose 37.25 ± 2.80 mg/m^2^; 10 patients with combined CT and CA, with 6-MP dose 39.62 ± 4.24 mg/m^2^. 6-MP dose had no significant difference between these three groups.

In this study, there were 10 patients with combined *NUDT15* c.415C>T (CT) and *ITPA* c.94C>A (CA) variants. We further evaluated the mixed effects of both *NUDT15* and *ITPA* variants on 6-MP tolerable dose. Twenty-seven patients had single heterozygotes *NUDT15* c.415C>T (CT), with 6-MP tolerable dose 33.56 ± 2.70 mg/m^2^. Thirty-two patients had single heterozygotes *ITPA* c.94C>A (CA), with 6-MP tolerable dose 37.25 ± 2.80 mg/m^2^. Ten patients had combined heterozygotes *NUDT15* c.415C>T (CT) and *ITPA* c.94C>A (CA), with 6-MP tolerable dose 39.62 ± 4.24 mg/m^2^. The 6-MP tolerable dose among these three groups had no significant difference (Figure 5).

## Discussion

With the development of next-generation sequencing techniques, genomic data have been incorporated into ALL risk classification, treatment, and prognostic system and greatly improved survival of pediatric ALL patients (18). Clinicians could reduce drug resistance, avoid adverse events, and improve overall survival based on pharmacogenomics. TPMT is the best example of application of pharmacogenomics to clinical practice. In 2019, the Clinical Pharmacogenetics Implementation Consortium updated 6-MP dose in a clinic setting based on *TPMT* genotype. However, differences in genotype distribution and frequency of *TPMT* alleles among different ethnic populations limit its predictive value. In this study, we focused on TPMT^*^3C, the most frequent *TPMT* genotype in China (19–21). In 149 pediatric ALL, only four TPMT^*^3C heterozygotes were found with allele frequency of 1.34%, a little lower than that in Chinese children with ALL (2.9%) reported by Zhou et al. (12), but higher than in Korean (0.6%) (22) and Indonesian pediatric ALL (0.95%) patients (23). Meanwhile, no significant association between TPMT^*^3C and 6-MP toxicities was found in our study, which was consistent with previous reports (23–25) but not compatible with another report from Chinese pediatric ALL by Zhou et al. (12). This discrepancy may be attributed to various factors, such as patient characteristics. In Zhou et al. study, the frequency of TPMT^*^3C is relatively high, so additional studies on a larger scale are warranted to make it clear in the future. In this study, the four TPMT^*^3C heterozygotes included three ethnic Han and one ethnic minority (ethnic Hani); the allele frequency of ethnic Han (1.3%) was compatible with that of other reports at 1.0% (19).

Considering the low frequency of *TPMT* polymorphism but comparable rates of 6-MP myelotoxicity in Asians, researchers have noted *NUDT15* polymorphisms as an important determinant factor for 6-MP myelotoxicity, especially *NUDT15* c.415C>T (rs116855232) (10, 26). Our study first described the frequency of *NUDT15* c.415C>T variant and association with 6-MP in pediatric ALL patients in this Yunnan province, a multiethnic region. In 149 pediatric ALL patients, 37 heterozygotes (CT) and three homozygotes (TT) were found with an allele frequency of 14.43%, consistent with that of another Chinese ALL group (15.7%) and other East Asia populations (9.8–16.8%) (Table 3), much higher than in European population (0.2–0.4%) (Table 3). In terms of 6-MP toxicity, we found a strong association between *NUDT15* c.415C>T with elevated risk of 6-MP-associated leucopenia (*p* = 0.008), but no association with hepatotoxicity (*p* = 0.37). In a clinic setting, 6-MP dose would be decreased for leucopenia or hepatotoxicity. In a further study, we found statistical significance *NUDT15* c.415C>T caused by 6-MP tolerable dose decrease (*p* = 0.021). In particular, the T allele was significantly correlated with 6-MP tolerable dose decreased. Compared with *NUDT15* c.415C>T wild (CC) and heterozygotes (CT), homozygote genotype (TT) was more intolerant to 6-MP, and 6-MP tolerable dose was decreased to 18.95 ± 3.95 mg/m^2^ (CC 39.80 ± 1.32 mg/m^2^, CT 35.20 ± 2.29 mg/m^2^). Considering different treatment protocols with different 6-MP doses in a maintenance period, most studies used 6-MP dose intensity. In this study, we used 6-MP tolerable dose to illustrate the treatment strength directly and found it different from other reports, such as Taiwan Chinese (TT 9.4 mg/m^2^, CT 30.7 mg/m^2^, and CC 44.1 mg/m^2^) (27) and other Chinese groups (TT 30.14 mg/m^2^, CT 41.92 mg/m^2^, and CC 47.12 mg/m^2^) (12). There may be various factors attributed to it, such as patient characteristics, clinician decision, complexity of pharmacogenomics, and medical level in different regions. These suggested that the precision therapy relied on not one factor but multiple factors; thus, more studies on a larger scale with different 6-MP regimens are needed to fully elucidate 6-MP individualized dose adjustment.

**Table 3:** Frequency of *NUDT15* c.415C>T (rs116855232) with ALL in different ethnicities.

**References**	**Ethnicity**	**Number of patients**	**Allele frequency (%)**
Yang et al. (11)	East Asian	61	9.8
	Hispanic	222	3.9
	European	205	0.2
	African	94	NA
Tanaka et al. (26)	Japanese	95	16.8
Liang et al. (27)	Taiwan Chinese	404	11.6
Buaboonnam et al. (28)	Thai	102	12.7
Zhou et al. (12)	Chinese	105	15.7
Lee et al. (22)	Korean	83	9.6
Moradveisi et al. (29)	Lebanon	136	0.4
	Kurdistan	74	NA

*ALL, acute lymphoblastic leukemia*.

A retrospective study showed reducing 6-MP starting dose based on *TPMT* polymorphisms reduced second malignant neoplasm (SMN) risk but increased relapse risk (30). In this study, we evaluated the risk of *NUDT15* c.415C>T on long-term survival of patients with ALL. Our results indicated that there was no significant difference of EFS between CC and CT/TT patients, which was consistent with report by Tanaka (26). But as Tanaka suggested, EFS probabilities appeared lower in patients with CT and TT genotypes. A larger-scale study with longer time followed up is needed.

ITPA is related to thioprine metabolism and also a potential predictor of 6-MP toxicity, but the clinical relevance of *ITPA* polymorphisms in 6-MP intolerance is still controversial (12, 31). In our study, frequency of *ITPA* c.94C>A was higher than that of *NUDT15* c.415C>T; 42 heterozygotes (CA) and seven homozygotes (AA) were found with allele frequency of 18.79%. We found no significant association with 6-MP toxicities, which was consistent with previous report in other Chinese pediatric ALL patients (12). Meanwhile, there were 10 patients with combined *NUDT15* c.415C>T (CT) and *ITPA* c.94C>A (CA) variants, but we did not discover any statistically significant differences of 6-MP dose between the *NUDT15* carriers, or *ITPA* carriers, or both carriers (Figure 5). In the future, a study of larger populations and additional other variants is needed to evaluate its effects on 6-MP.

Our study also had some limitations. First, we only focused on one variant of each gene and missed some other potential genetic polymorphisms. Second, our study had a small sample size, especially the small number of ethnic minorities, resulting in a low power to detect differences. Third, we only discovered a significant relationship of *NUDT15* c.415C>T with neutropenia and did not elaborate the underlying mechanism on how this variant influenced the toxicity of 6-MP. Valerie et al. found that *NUDT15* c.415C>T did not affect enzymatic activity but negatively influenced protein stability, which thus lost supportive intramolecular bonds and caused rapid NUDT15 proteasomal degradation, which finally induced DNA damage checkpoint and cancer cell death by 6-thioguanine (32). Moreover, 6-MP tolerable dose was adjusted by many factors, such as 6-MP toxicity, the combined use of MTX, and patient compliance, but we only focused on 6-MP toxicity. Further studies will focus on these questions to achieve 6-MP precise treatment in a clinic setting.

## Conclusions

We first elucidated the variant frequencies of TPMT^*^3C (rs1142345), *NUDT15* c.415C>T (rs116855232), and *ITPA* c.94C>A (rs1127354) in pediatric ALL from Yunnan province, a multiethnic region in China. Among these genetic variants, we found that *NUDT15* c.415C>T (rs116855232) could predict 6-MP toxicity and intolerance during ALL maintenance therapy, but the 6-MP tolerable dose in our data was not so consistent with that of other reports of Asian population. We also found no statistical difference of EFS in ALL patients with *NUDT15* variants. Further clinical studies in larger scale with more genetic polymorphisms are required to develop better and precise treatment strategies in ALL patients.

## Data Availability Statement

The original contributions presented in the study are included in the article/Supplementary Material, further inquiries can be directed to the corresponding authors.

## Ethics Statement

The studies involving human participants were reviewed and approved by Ethics Committee of children's hospital affiliated to Kunming Medical University. Written informed consent to participate in this study was provided by the participants' legal guardian/next of kin.

## Author Contributions

XT designed the study, analyzed the patient data, and concluded the value of the research. XM performed the research and was a major contributor in writing the manuscript. RY, GS, YZ, and CY collected the patient data. CF, YW, TC, LL, and JG did the follow-up work. All authors contributed to the article and approved the submitted version.

## Funding

This work was supported by the National Natural Science Foundation of China (No. 81760032) to XT.

## Conflict of Interest

The authors declare that the research was conducted in the absence of any commercial or financial relationships that could be construed as a potential conflict of interest.

## Publisher's Note

All claims expressed in this article are solely those of the authors and do not necessarily represent those of their affiliated organizations, or those of the publisher, the editors and the reviewers. Any product that may be evaluated in this article, or claim that may be made by its manufacturer, is not guaranteed or endorsed by the publisher.
